# The pair ceramide 1-phosphate/ceramide kinase regulates intracellular calcium and progesterone-induced human sperm acrosomal exocytosis

**DOI:** 10.3389/fcell.2023.1148831

**Published:** 2023-03-31

**Authors:** Cintia C. Vaquer, Laila Suhaiman, Martín A. Pavarotti, Rodolfo J. Arias, Anahí B. Pacheco Guiñazú, Gerardo A. De Blas, Silvia A. Belmonte

**Affiliations:** ^1^ Instituto de Histología y Embriología de Mendoza (IHEM) “Dr. Mario H. Burgos”, CONICET, Universidad Nacional de Cuyo, Mendoza, Argentina; ^2^ Facultad de Ciencias Médicas, Universidad Nacional de Cuyo, Mendoza, Argentina; ^3^ LaTIT. Área Farmacología, Facultad de Ciencias Médicas, Universidad Nacional de Cuyo, Mendoza, Argentina

**Keywords:** human sperm, ceramide 1-phosphate/ceramide kinase, acrosome exocytosis, calcium channels, sphingolipids

## Abstract

Before fertilization, spermatozoa must undergo calcium-regulated acrosome exocytosis in response to physiological stimuli such as progesterone and zona pellucida. Our laboratory has elucidated the signaling cascades accomplished by different sphingolipids during human sperm acrosomal exocytosis. Recently, we established that ceramide increases intracellular calcium by activating various channels and stimulating the acrosome reaction. However, whether ceramide induces exocytosis on its own, activation of the ceramide kinase/ceramide 1-phosphate (CERK/C1P) pathway or both is still an unsolved issue. Here, we demonstrate that C1P addition induces exocytosis in intact, capacitated human sperm. Real-time imaging in single-cell and calcium measurements in sperm population showed that C1P needs extracellular calcium to induce [Ca^2+^]i increase. The sphingolipid triggered the cation influx through voltage-operated calcium (VOC) and store-operated calcium (SOC) channels. However, it requires calcium efflux from internal stores through inositol 3-phosphate receptors (IP_3_R) and ryanodine receptors (RyR) to achieve calcium rise and the acrosome reaction. We report the presence of the CERK in human spermatozoa, the enzyme that catalyzes C1P synthesis. Furthermore, CERK exhibited calcium-stimulated enzymatic activity during the acrosome reaction. Exocytosis assays using a CERK inhibitor demonstrated that ceramide induces acrosomal exocytosis, mainly due to C1P synthesis. Strikingly, progesterone required CERK activity to induce intracellular calcium increase and acrosome exocytosis. This is the first report, implicating the bioactive sphingolipid C1P in the physiological progesterone pathway leading to the sperm acrosome reaction.

## Introduction

Sphingolipids are ubiquitous components of eukaryotic cells that accomplish vital roles in cell growth, death, and survival. Ceramide 1-phosphate (C1P) is a bioactive sphingolipid metabolite considered a signaling lipid. Ceramide kinase (CERK), phosphorylates ceramide to ceramide C1P in mammalian cells (Presa et al., 2020). This enzyme was initially characterized as a calcium-stimulated lipid kinase in synaptic vesicles (Bajjalieh et al., 1989). The C1P/CERK pathway has been involved in the regulation of essential pathophysiological functions such as phagocytosis and inflammation (Presa et al., 2016).

C1P regulates directly the activities of the cytosolic phospholipase A2 (cPLA2α) (Nakamura et al., 2006), sphingosine kinase (SK1) (Nishino et al., 2019; Hori et al., 2020), and tumor necrosis factor *a*-converting enzyme (Lamour et al., 2011). Further, C1P governs many cellular responses like cell migration and exocytosis by acting directly or indirectly *via* cell signaling involving putative receptors (Mitsutake et al., 2004; Presa et al., 2016; Hori et al., 2020).

The exocytosis, as the last step of the secretory pathway, is a precisely ordered and calcium-controlled process entailing tethering, docking, and fusion of vesicles to the plasma membrane for cargo release (Zhang and Staiger, 2021). Exocytosis is regulated by sphingolipids in PC12 cells. E.g., dopamine release by calcium ionophores is mediated by the production of ceramide from sphingomyelin (Jeon et al., 2005) and extracellular application of ceramide caused exocytosis in PC12 cells (Tang et al., 2007). C1P/CERK pathway regulates dopamine transporters recycling (Won et al., 2018). Some studies have identified the role of CERK in arachidonic acid release in a lung epithelial cell line (Pettus et al., 2003; Pettus et al., 2004; Subramanian et al., 2005).

Mitsutake et al. (2004) described that the enzyme CERK is a mediator of calcium-dependent mast cell degranulation (Mitsutake et al., 2004). Hewson et al. (2011) demonstrated that C1P induced histamine and PGD2 release in mast cells. CERK was involved in the exocytosis of these mediators and some molecules of the signaling pathway have been identified (Hewson et al., 2011).

The acrosome is the main calcium store of the male gamete. The exocytosis of the sperm granule, termed acrosome reaction (AR), is crucial for mammal fertilization. It involves complex biological changes that culminate with the release of the granule content and the plasma membrane reorganization (Belmonte et al., 2016). The last step enables sperm to interact and fuse with the oocyte (Inoue et al., 2011; Bianchi et al., 2014; Bianchi and Wright, 2020). Innumerable physiological events involved in the AR remain elusive, although it is known that during the AR there is an increase in the intracellular Ca^2+^ concentration ([Ca^2+^]i) (Darszon et al., 2005; Stival et al., 2016). Therefore, orchestrated [Ca^2+^]i augments are necessary for the AR to occur. Briefly, the canonical mechanism described proposes that a temporary calcium entry through plasma membrane channels induces inositol 3-phosphate (IP_3_) synthesis and IP_3_R activation in the granule elicits calcium efflux from the acrosome calcium reservoir. The stromal interaction molecule, STIM, senses the acrosome calcium content decrease and bestows calcium store-depletion into the opening of Orai channels at the PM (Darszon et al., 2011; Sosa et al., 2015; Sosa et al., 2016). This event triggers a prolonged and continuous calcium increase due to SOC channels opening (Aldana et al., 2021). Abundant evidence exists for another functional calcium store present at the neck piece: the redundant nuclear envelope (RNE). Its membrane contains IP_3_R and ryanodine receptors (RyR) (Harper and Publicover, 2005; Harper et al., 2006; Suarez, 2008). It is necessary to highlight the presence of CatSper (calcium channel of sperm), present at the flagellum principal piece. It is a voltage- and pH-dependent gated calcium channel, activated by Pg and required for male fertility (Kirichok et al., 2006; Lishko et al., 2011).

Our laboratory has elucidated the signaling cascades accomplished by different sphingolipids during sperm acrosomal exocytosis (Suhaiman et al., 2010; Vaquer et al., 2020). We have not only demonstrated the presence and activity of some enzymes of the sphingolipid metabolism in a terminal cell like the human sperm but we defined how some of these enzymes and lipids regulate exocytosis. The bioactive sphingolipid, sphingosine 1-phosphate (S1P), triggers AR by interacting with Gi-coupled receptors and activating extracellular calcium influx through voltage and store-operated calcium channels as well as efflux from intracellular stores through IP_3_-sensitive calcium channels. We showed for the first time that human spermatozoa produce S1P, which induces exocytosis through an autocrine/paracrine action (Suhaiman et al., 2010). Recently, we established the effect of ceramide, a metabolic precursor of S1P, during acrosomal exocytosis. Both, exogenous and endogenous ceramide induces AR by raising [Ca^2+^]i during the first 40 s activating calcium influx through CatSper and SOC channels. The sphingolipid also promotes calcium efflux through RyR from RNE. A striking finding was that even though ceramide is a precursor of S1P its action is exerted in an S1P-independent manner (Vaquer et al., 2020). However, whether ceramide induces exocytosis by itself or activates the CERK/C1P pathway, or whether both mechanisms are related is still an unresolved issue. In the present study, we demonstrated that the pair C1P/CERK regulates intracellular calcium concentration and induces human sperm acrosomal exocytosis. Further, the CERK activity and thus C1P synthesis are required for progesterone-elicited AR. This research is unique in determining the effect of C1P during acrosomal exocytosis and its involvement in the progesterone (Pg) physiological pathway.

## Materials and methods

### Reagents

Spermatozoa were maintained in Human Tubal Fluid medium (HTF), which contains 0.35 g/L KCl, 5.94 g/L NaCl, 0.05 g/L KH_2_PO_4_, 0.05 g/L MgSO_4_·7H_2_O, 0.3 g/L CaCl_2_ .2H_2_O, 2.1 g/L NaHCO_3_, 0.51 g/L D-glucose, 2.39 g/L Na lactate, 0.036 g/L Na pyruvate, 0.05 g/L streptomycin, 0.06 g/L penicillin, 0.01 g/L phenol red. Sphingosine kinase inhibitor (SKI), 2-aminoethoxydiphenylborate (2-APB), and adamantane-1-carboxylic acid (2-benzoylamino-benzothiazol-6-yl)amide, N-[2-(benzoylamino)-6-benzothiazolyl]-Tricyclo [3.3.1.13,7]decane-1-carboxamide (NVP-231) were from Calbiochem (MERCK Argentina). C2 Ceramide-1-Phosphate (d18:1/2:0) and C6-ceramide were from Avanti Polar Lipids, Inc. (Alabaster, AL, United States). A23187 and dantrolene were from Alomone (Alomone Labs. Ltd. Jerusalem, Israel). TLC aluminum sheets silica gel 60 were from MERCK KGaA (Darmstadt, Germany). Verapamil, 4-methyl-4′-[3,5-bis (trifluoromethyl)-1H-pyrazol-1-yl]-1,2,3-thiadiazole-5-carboxanilide (YM-58483) and 1-[b-[3-(4-methoxyphenyl)propoxy]-4-methoxyphenethyl]- 1H-imidazole (SKF-96365), xestospongin C, and progesterone were from Sigma (Sigma-Aldrich Argentina SA). Albumin and fluorescein-isothiocyanate-coupled Pisum sativum lectin were from ICN (Eurolab SA, Buenos Aires, Argentina). Horseradish peroxidase- and Cy^TM^3-conjugated goat anti-rabbit antibodies were from Jackson ImmunoResearch (West Grove, PA). Anti-CERK (Abcam). N-Lauroyl-D-erythro-sphingosine, ruthenium red, ionomycin, 1,2-bis amino phenoxy)ethane-N,N,N0,N0-tetraacetic acid (BAPTA), and glycine, 4-(6-Acetoxymethoxy-2,7-dichloro-3-oxo-9-xanthenyl)-4′-methyl-2,2′(ethylenedioxy) dianiline- N,N,N′,N′-tetraacetic acid tetrakis (acetoxymethyl) ester (Fluo-3 AM) were from Molecular Probes (Invitrogen Argentina). Ted Pella Inc. provided the electron microscopy supplies. Recombinant streptolysin O (SLO) was from Dr. Bhakdi (University of Mainz, Mainz, Germany). Any other reagents were purchased from Sigma-Aldrich™, Tecnolab, or Genbiotech.

### Ethics statement and human sperm preparation

Healthy male donors provided ejaculates by masturbation, after at least 48 h of sexual abstinence. We used only semen samples that accomplished the World Health Organization (WHO, 2021) specifications, for the experiments shown here. Data collection adheres to the guidelines established in Argentina (ANMAT 5330/97) and the International Declaration of Helsinki. All donors signed an informed consent according to supply semen samples. The protocol for semen manipulation was accepted by the Ethics Committee of the School of Medicine, National University of Cuyo.

After semen liquefaction (30–60 min at 37°C), motile sperm were retrieved after a swim-up procedure for 1 h at 37°C in HTF. We adjusted sperm concentration to 10^6^ cells/mL and further incubated for 2 h under non-capacitating (HTF, 5% CO_2_/95% air, 37°C) or 2–5 h under capacitating (HTF supplemented with 0.5% BSA, 5% CO_2_/95% air, 37°C) conditions. When permeabilized sperm are required, we washed capacitated cells and resuspended them in cold PBS with 3 U/mL SLO, and incubated for 15 min at 4°C. We washed sperm twice with PBS and suspended in ice-cold sucrose buffer (20 mM Hepes-K, 0.5 mM EGTA, 250 mM sucrose, pH 7.0) with 2 mM DTT. Then, we follow the procedures for AR assays, Western blot, enzyme activities, and calcium measurements.

### Sperm acrosome exocytosis measurements

We incubated capacitated or non-capacitated sperm suspensions consecutively with inhibitors and stimulants as described in the legends to figures. We kept the cells for 10–15 min at 37°C after each addition. The acrosome status was assessed by FITC-PSA (25 μg/mL in PBS) staining as described in Mendoza et al., 1992 (Mendoza et al., 1992) and as detailed in our publications (Suhaiman et al., 2010; Belmonte & Suhaiman, 2012; Vaquer et al., 2020; Suhaiman et al., 2021). We scored at least 300 cells using a Nikon Optiphot II microscope equipped with epifluorescence optics. In each experiment, negative controls (no stimulation) and positive controls (stimulated with A23187 or progesterone) were included. For each experiment, the data were normalized by subtracting the number of reacted spermatozoa in the negative control from all values and expressing the result as a percentage of the acrosome reaction observed in the positive control (100%). The actual percentages of reacted sperm for negative and positive controls ranged between 6% and 25% and 25% and 40%, respectively. Experiments in which the difference between positive and negative controls was less than 10% were discarded. The average difference between positive and negative controls was 14%.

We performed one-way ANOVA to analyze the data. Conditions used for data normalization were not included in the evaluation (0% and 100%). Dunnett *post hoc* test or Tukey-Kramer were utilized to compare the means with a control condition. When specified, we utilized a Student’s t-test or Newman-Keuls Multiple Comparison Test. We considered significant differences at the *p* > 0.05 level.

### Indirect immunofluorescence

We fixed capacitated sperm (5 × 10^6^ cells) in 2% paraformaldehyde for 15 min at RT and resuspended them in 100 mM glycine in PBS. We attached sperm to poly-L-coated, coverslips. Next, we permeabilized the plasma membrane with 0.1% Triton X-100 in PBS for 10 min at RT, and washed twice with 0.1% polyvinylpyrrolidone in PBS for 1 h at 37°C. To block non-specific marks, we incubated the sample with 5% BSA in PBS/PVP for 1 h at 37°C. Then, we incubated the cells with anti-CERK (Abcam, 20 μg/mL) for 1 h at RT in a moisturized chamber. As a second antibody, we used Cy^TM^3-conjugated anti-rabbit in BSA 1%/PBS/PVP. Then, we fixed/permeabilized the acrosome membrane with cold methanol (−20°C) for 20 s and stained the cells with FITC-PSA as described in here *Sperm acrosome exocytosis measurements*. Sperm were observed by confocal microscopy (Olympus FluoViewTM FV1000 confocal microscope, Olympus, Argentina), with the FV10-ASW software. Images were processed with MetaMorph, ImageJ, and Corel Draw. The protocol is detailed in our previous publications (Suhaiman et al., 2010; Belmonte and Suhaiman, 2012; Suhaiman et al., 2021).

### SDS-PAGE and Western blot

We electrophoresed proteins on 8% Tris-glycine SDS gels and transferred them to nitrocellulose membranes. We blocked non-specific reaction with 3% BSA in washing buffer (PBS pH 7.4, 0.1% Tween 20) for 1 h at RT. Blots were incubated with anti-CERK (Abcam, 0.1 μg/mL) for 2 h at RT. As a secondary antibody, we utilized horseradish peroxidase-conjugated goat anti-rabbit IgG (0.1 μg/mL in washing buffer) with 1 h incubation at RT. We detected the protein with a chemiluminescence system (Kalium Technologies SRL, Argentina) on a Luminescent Image Analyzer LAS-4000 (Fujifilm, Tokyo, Japan).

### Transmission electron microscopy

Capacitated sperm were treated with 10 μM C1P or 10 μM A23187 (used as a positive control), and incubated for 15 min at 37°C. As a negative control, we included a sample without any treatment. We washed sperm twice in PBS and fixed them in 2.5% v/v glutaraldehyde in 0.1 M sodium cacodylate buffer for 2 h at 10°C. We centrifuged the samples for 30 s at 10,000 rpm. The sperm pellets obtained were further fixed in 1% OsO_4_ for 1 h at RT. We used graded acetone series to dehydrate cells and embedded them in low-viscosity epoxy resin. The resin was polymerized at 70°C for 48 h. Using a diamond knife, we obtained ultrathin sections in an ultramicrotome (Ultracut R; Leica, Austria) with an interference color grey. We collected ultrathin sections on 200-mesh copper grids and stained them with uranyl acetate and lead citrate as described in (Belmonte et al., 2005). We observed the samples under the electron microscope Zeiss 900 (Zeiss, Jena, Germany) at 80 kV. Micrographs were obtained with a Gatan Orius SC1000 (model 832) charge-coupled device. The samples were processed by A. Morales, Ph.D. and P. López, MS from the STAN: ST3371 of TEM and SEM samples preparation, IHEM-CONICET-UNCuyo. At least 100 cells per condition were scored and sorted the acrosomal patterns as intact, swollen (swollen and waving), and reacted (lost and vesiculated acrosomes). Newman-Keuls Multiple Comparison Test allowed us to analyze all these data by using the GraphPad program Prism 5. Significant differences were considered at the *p* < 0.05 level.

### Tetanus toxin conjugation with cell-penetrating peptides

We prepared the protein as detailed in Mayorga et al. (2020) (Mayorga et al., 2020). In short, we transformed the plasmid’s DNA coding 6His-light chain of tetanus toxin (pQE3plasmid, Qiagen) into *E. coli* XL-1Blue (Stratagene). We induced protein expression with 0.2 mM isopropyl 1-thio-D-galactopyranoside, ON at 20°C. We purified the 6His-tagged proteins according to the QIAexpressionist (www.qiagen.com). We used Trilinks Biotechnology Kits and followed the manufacturer’s instructions, to conjugate the CPP peptides (KRRRRRRRRRC) and tetanus toxin. SDS-PAGE analysis allowed us to validate purity.

### Sphingosine kinase activity measurement

We measured sphingosine kinase 1 (SK1) activity in permeabilized sperm as described in Suhaiman et al. (2010) (Suhaiman et al., 2010). We washed 50 × 10^6^ spermatozoa with cold PBS, after permeabilization with SLO (Pelletan et al., 2015), and resuspended them in SK1 buffer (20 mM Tris-HCl, pH 7.4, 1 mM EDTA, 0.5 mM deoxypyridoxine, 15 mM NaF, 1 mM *ß*-mercaptoethanol, 1 mM sodium orthovanadate, 40 mM *ß*-glycerophosphate, 0.4 mM phenylmethylsulfonyl fluoride, 10% glycerol, 0.5% Triton X-100, 10 mM MgCl_2_, and Complete protease inhibitors). Then, we incubated sperm in 100 μL of SK1 buffer: sphingosine (50 μM, delivered in 4 mg/mL fatty acid-free bovine serum albumin) and [γ-^32^P]ATP (5 μCi) for 30 min at 37°C. We used HeLa cells incubated with sphingosine as a positive control (30 μg of proteins) and without sphingosine addition as a negative control. When indicated, sperm were incubated for 15 min at 37°C with 1 μM SKI (specific Sphingosine Kinase Inhibitor). Subsequently, we added 10 μM C6-ceramide (C6), washed it with cold PBS, and suspended it in SK1 buffer. We stopped the reaction with 10 μL of 1 N HCl and 400 μL of chloroform/methanol/HCl (100:200:1, v/v). Then, we added 120 μL of chloroform and 120 μL of 2 M KCl. We centrifuged the samples at 3,000 g for 10 min. We transferred 200 μL of the organic phase to new glass tubes and dried them. We resuspended the samples in chloroform/methanol/HCl (100:100:1, v/v). Then we resolved the lipids on TLC plates utilizing 1-butanol/methanol/acetic acid/water (8:2:1:2, v/v) as a solvent system and observed by autoradiography.

### Ceramide kinase (CERK) activity measurement

We determined CERK as detailed in Tada et al. (2010) (Tada et al., 2010) and introduced the modifications described for sperm in Vaquer et al. (2020) (Vaquer et al., 2020). In brief, we incubated 100 × 10^6^ cells with 10 μM C12-NBD dissolved in 4 mg/mL fatty acid-free BSA (FAF-BSA) for 1 h at 37 °C. We kept sperm in the dark to prevent NBD degradation. After that, sperm were washed with HTF/0.4% FAF-BSA. Phosphatase inhibitors were added to the mixture: 1 mM sodium orthovanadate and 15 mM NaF. When indicated, we incubated sperm with 200 nM NVP-231 (CERK inhibitor) for 20 min at 37°C. After that, we added A23187 (10 μM) and treated the cells for 20 min. We ended the reaction by adding 500 μL methanol, 250 μL chloroform, and mixed-up. Afterward, we incorporated 500 μL of chloroform and 250 μL of water. Sperm were centrifuged at 600 *g* for 10 min. In the organic phase, we observed the C12-NBD (data not shown), and in the aqueous phase, two bands appear corresponding to lauric acid-NBD, produced due to ceramidase activity, as shown in our previous publication (Vaquer et al., 2020) and another band that matches with the retention factor (Rf) of C1P. Lipids were resolved on TLC plates by using 1-butanol/acetic acid/water (3:1:1, v/v/v) and visualized by fluorescence with LAS-4000 (Fujifilm (λEx: 498 nm - λEm: 522 nm). A semiquantitative evaluation of the spots was performed with ImageJ.

### [Ca^2+^]i measurements in single-cell

Sperm (10 × 10^6^) were loaded with Fluo-3 AM. Male gametes were immobilized on poly-L-lysine-coated coverslips, mounted on a chamber, and placed on the stage of an inverted microscope (Eclipse TE300 Nikon). Fluo-3 AM was excited with a stroboscopic LED-based fluorescence illumination system as detailed in (Pocognoni et al., 2013). We compiled images (7 frames/min). A filter with the following bandwidths: excitation 450–490 nm, dichroic mirror 505 nm, and emission 520–560 nm were used. We utilized a Plan Fluor 40×/0.6 Nikon objective and compiled images using NIS Element software (Nikon). C1P was microinjected to the sperm suspension (injection speed: 73 nL per second). Intracellular calcium was measured once ionomycin was added to calibrate the maximal response. We performed fluorescence measurements as detailed in Suhaiman et al. (2010) (Suhaiman et al., 2010). Fluorescence data were processed offline with ImageJ. We normalized raw intensity values imported with the following equations: a) (F/Fo) − 1. F is the fluorescence intensity at time t and Fo is the mean of F taken during the control period. We graphed all the results of (F/Fo) −1 vs. time. %Δ(F/Fo) −1 is the difference between the fluorescence before stimulation and the maximal fluorescence obtained after the addition of C1P. We analyzed and graphed the data with GraphPad Prism 5 (four independent experiments).

### Calcium measurements in cell population

We loaded 5–10 × 10^6^ sperm/mL of motile sperm with the permeable Fluo-3 AM dye (2 μM). Afterwards, we washed sperm and suspended them in nominally calcium-free (∼1 μM) medium (10 mM Hepes-Na, 4 mM KCl, 120 mM NaCl, 15 mM NaHCO_3_, 5 mM D-glucose, 1 mM sodium pyruvate, 10 mM lactic acid, 1 mM MgCl_2_, pH 7.4). We used cuvettes at 37°C to perform sperm fluorescence measurements. At the times indicated, we added 15 μM Pg or 10 μM C1P to the samples. We recorded Fluo-3 AM fluorescence (λEx = 505, λEm = 525 nm) in an Aminco Bowman II spectrofluorometer. To assess [Ca^2+^]i movements, we chelated extracellular calcium with 0.75 mM EGTA and 0.5 mM Ca^2+^ to the medium (calculated by MAXCHELATOR, https://web.stanford.edu/∼cpatton/, Chris Patton, Stanford University, Stanford, CA, United States) resulting in a final extracellular [Ca^2+^] ≤ 100 nM. Cells were loaded with Fluo-3 AM, washed once, and suspended in buffer (0.75 mM EGTA, 0.5 mM Ca^2+^, 10 mM Hepes-Na, 120 mM NaCl, 15 mM NaHCO_3_, 5 mM D-glucose, 4 mM KCl, 1 mM MgCl_2_, 10 mM lactic acid, 1 mM sodium pyruvate, pH 7.4). When indicated, we added to sperm suspension 15 μM Pg or 10 μM C1P. We recorded Fluo-3 fluorescence as stated above. To test which calcium channels are involved in the calcium increment induced by C1P, we run experiments using different calcium channel blockers in a media with a final [Ca^2+^] concentration of 2 mM. Cells loaded with Fluo-3 AM were incubated for 10 min with 200 μM lanthanum, 100 μM Verapamil, 1 μM XC, 100 μM 2-APB, 1 mM YM-58483, 50 mM SKF-96365, 1 μM NNC 55–0396 (NNC), 100 μM dantrolene, or 20 nM Ruthenium red (RR), previous the addition of C1P (10 μM). We collected the data for 600 s at a frequency of 4 Hz (2 fps). We calibrated the maximal [Ca^2+^]i response after Triton X-100 (0.1%) addition. We used five different batches of sperm to perform independent measurements. We utilized the Tukey-Kramer *post hoc* test for pairwise comparisons. We considered significant differences at the *p* ≥ 0.05 level.

## Results

### C1P induces exocytosis in human sperm capacitated “*in vitro*”

First, we started by testing if C1P was able to directly accomplish the AR. Considering that C1P acts, as an extracellular and intracellular signaling molecule, we performed experiments using intact and plasma membrane-permeabilized human spermatozoa.

**FIGURE 1:**
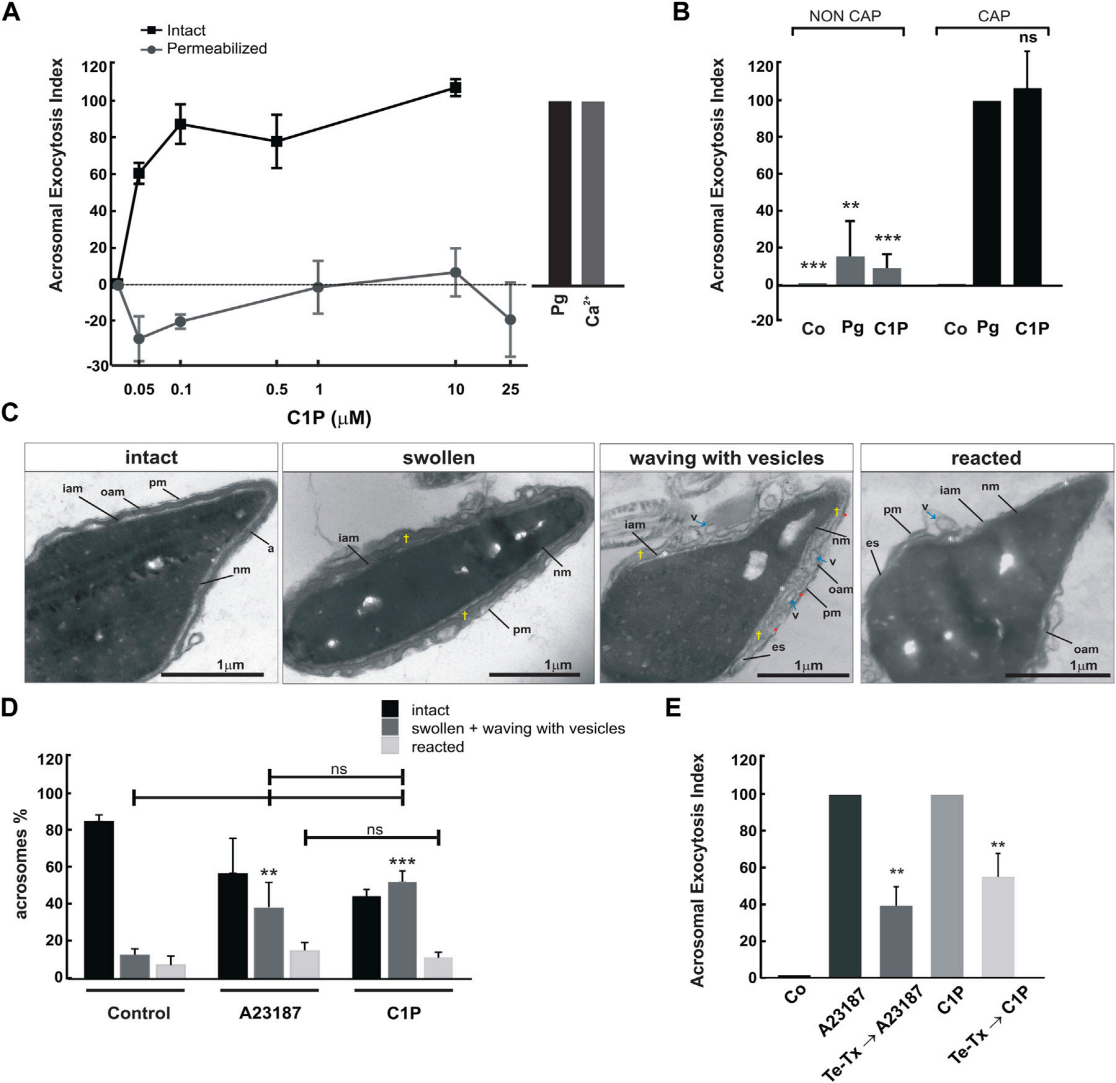
C1P triggers acrosomal exocytosis in capacitated intact human spermatozoa. **(A)** After swim-up in HTF (5 mg/ml bovine serum albumin) at 37°C, 5% CO_2_, sperm were incubated for at least 3 h under capacitating conditions. Intact and controlled plasma membrane SLO-permeabilized sperm were treated with increasing concentrations of C1P (0–10 μM) for 15 min at 37°C in 5% CO_2_. As a positive control, permeabilized spermatozoa were stimulated with 10 μM free Ca^2+^ (gray bar) and intact sperm with 15 μM Pg (black bar). Sperm were fixed and acrosomal exocytosis was evaluated by FITC-PSA binding with at least 300 cells per condition scored. The data represent the mean ± S.E. from three to five independent experiments. **(B)** An aliquot of a sperm sample was subjected to swim-up in HTF medium under non-capacitating conditions (37°C, 5% CO_2_, without bovine serum albumin). Another aliquot was processed in capacitating conditions: swim-up in HTF (supplemented with 5 mg/ml bovine serum albumin, 37°C, 5% CO_2_). Cells recovered from swim-up were incubated for an additional 3 h under non-capacitating or capacitating conditions as indicated. Sperm were treated or not (control, Co) with 15 μM progesterone (Pg) or 10 μM C1P for 15 min at 37°C 5% CO_2_. Acrosomal exocytosis was evaluated as explained under “Materials and Methods”. The data represent the mean ± S.E. of at least four independent experiments. The means of groups NON CAP and CAP were compared with the corresponding control (Pg) using Dunnett’s test and classified as non-significant (ns, *p* < 0.05), or significant (**, *p* < 0.01; ***, *p* < 0.001). **(C)** Capacitated sperm were incubated for 15 min at 37°C with 10 μM C1P or 10 μM A23187 and processed as described in “Materials and Methods” for electron microscopy. Transmission electron micrographs of spermatozoa after C1P treatment showing different morphological stages: intact, swollen + waving with vesicles and reacted. pm: plasma membrane; oam: outer acrosomal membrane; iam: inner acrosomal membrane; nm nuclear membrane; es: equatorial segment; v: vesicle; †: swollen acrosome. Scale bars, 1 μm. **(D)** Quantification of the percentage of intact, swollen (swollen and waving with vesicles) and reacted sperm (lost acrosomes). The means of groups swollen plus waving with vesicles were compared with the corresponding control s, *p < 0.05; ns, *p* > 0.05 (Newman-Keuls Multiple Comparison Test). **(E)** Capacitated sperm were incubated with 1.5 μM recombinant cell-permeant light chain of Tetanus toxin (Te-Tx). After that, the AR was stimulated with 10 μM C1P or 10 μM A23187 for 15 min. Sperm was fixed and acrosomal exocytosis was evaluated by FITC-PSA binding with at least 300 cells scored.

First, we challenged intact spermatozoa with increasing C1P concentrations (from 0 to 10 μM) and measured the exocytotic response. Exogenous C1P triggered exocytosis in a dose-dependent way (Figure 1A) achieving a maximum at 0.1 μM. Even when C1P concentration was increased, the percentage of reacted cells remained in the same range. C1P elicited the acrosome exocytosis as effectively as Pg, the physiological inducer of the AR, used as a positive control (Figures 1A,B). It is worth highlighting that C1P did not induce exocytosis in permeabilized sperm (Figure 1A). As shown in Figure 1A (Ca^2+^), permeabilized cells treated with calcium (control) underwent exocytosis. Both, C1P 0.1 and 10 μM did not affect sperm viability assessed by using 0.1% eosin to stain dead cells (control, 92.6% ± 1.10; 0.1 μM C1P, 88.2% ± 2.98; 10 uM C1P 89.7% ± 1.73; mean ± S.E.).

Human sperm are not able to fertilize straight away after ejaculation. They acquire this ability during a lapse of permanence in the female reproductive tract. There, sperm undergo numerous complex physiological changes called capacitation. Sperm capacitation can be accomplished *in vitro* by incubating spermatozoa in artificial media mimicking the composition of the fluids present in the female reproductive tract. The exocytic response of human sperm to progesterone is a hallmark of capacitation (Belmonte et al., 2005; Mayorga et al., 2020). To elucidate if C1P requires sperm capacitation to induce exocytosis we performed AR experiments by incubating male gametes under capacitating (HTF media supplemented with 5 mg/mL BSA) or non-capacitating conditions (HTF without BSA). After 3 h incubation, we challenged the cells with 10 μM C1P. As shown in Figure 1B, capacitated sperm underwent AR when treated with C1P. In contrast, non-capacitated cells were not able to respond to the sphingolipid stimulus (non-cap, gray bars). We used 15 μM Pg to determine the capacitation status of the gametes (Belmonte et al., 2005; Muratori et al., 2008).

Handling lipids is tricky given that when added to the culture media they can be embedded in membranes affecting directly their integrity and/or function. Therefore, we ran various controls. First, we evaluated the physiological behavior of human sperm after C1P treatment. Swelling of secretory vesicles is a previous step occurring before the fusion of the granules with the plasma membrane (Finkelstein et al., 1986). The acrosome granule swelling was observed by electron microscopy in human sperm that have started exocytosis before they completely lose their acrosomes (Zanetti and Mayorga, 2009). Upon AR stimulation, the outer acrosome membrane (OAM) waves and the invagination edges encounter the plasma membrane. Both membranes form tight appositions finally stabilized by the assembly of SNAREs. Calcium rise leads to membrane fusion, the opening of fusion pores, hybrid vesicles (mixed composition containing OAM and plasma membrane), and acrosome content release (Suhaiman et al., 2021).

To discard the possibility of acrosome loss due to C1P-induced membrane destabilization, we searched for possible ultrastructural changes by TEM in human sperm after challenging them with C1P. Then, we incubated sperm for 15 min with 10 μM of C1P or the calcium ionophore, A23187. The percentage of swollen plus waving acrosomes increased up to ∼50% compared to the control (Figures 1C,D). Our data provide direct evidence indicating that C1P can induce the intermediate step necessary for sperm AR, the granule swelling. Further, this result allows us to rule out membrane disruption. The sperm AR is a synchronized process occurring once in the gamete life. It depends on neurotoxin-sensitive SNAREs activation. Calcium sparks tangled signaling in spermatozoa that ultimately disassembles neurotoxin-resistant cis and triggers toxin-sensitive loose trans-SNARE complexes assembly. Acrosome exocytosis requires all these steps to proceed (De Blas et al., 2005) (Lopez et al., 2012; Pelletan et al., 2015). To elucidate if C1P-induced exocytosis is a physiological process that requires functional SNAREs to proceed, we cleaved VAMP2 with a tool developed in the laboratory before adding the lipid. We utilized a recombinant Tetanus toxin (Te-Tx) coupled to a cell-penetrating peptide (Mayorga et al., 2020). This peptide permits plasma membrane translocation and toxin delivery to the cytosol. To assess our hypothesis, we first incubated human spermatozoa with the permeable Te-Tx light chain and after that, the sperm AR was stimulated with 10 μM C1P for 15 min. Te-Tx cleaved VAMP2 and significantly inhibited both, the calcium ionophore (A23187) and C1P-triggered AR (Figure 1E). This last result demonstrates that C1P requires the SNAREs assembly to induce the AR.

### C1P-triggers extracellular calcium influx leading to sperm acrosomal exocytosis

Hence, if C1P is inducing regulated exocytosis, we assumed that the sphingolipid should be increasing intracellular calcium. Then, we tested whether C1P addition to the sperm media was able to induce an [Ca^2+^]i augment in live cells.

**FIGURE 2:**
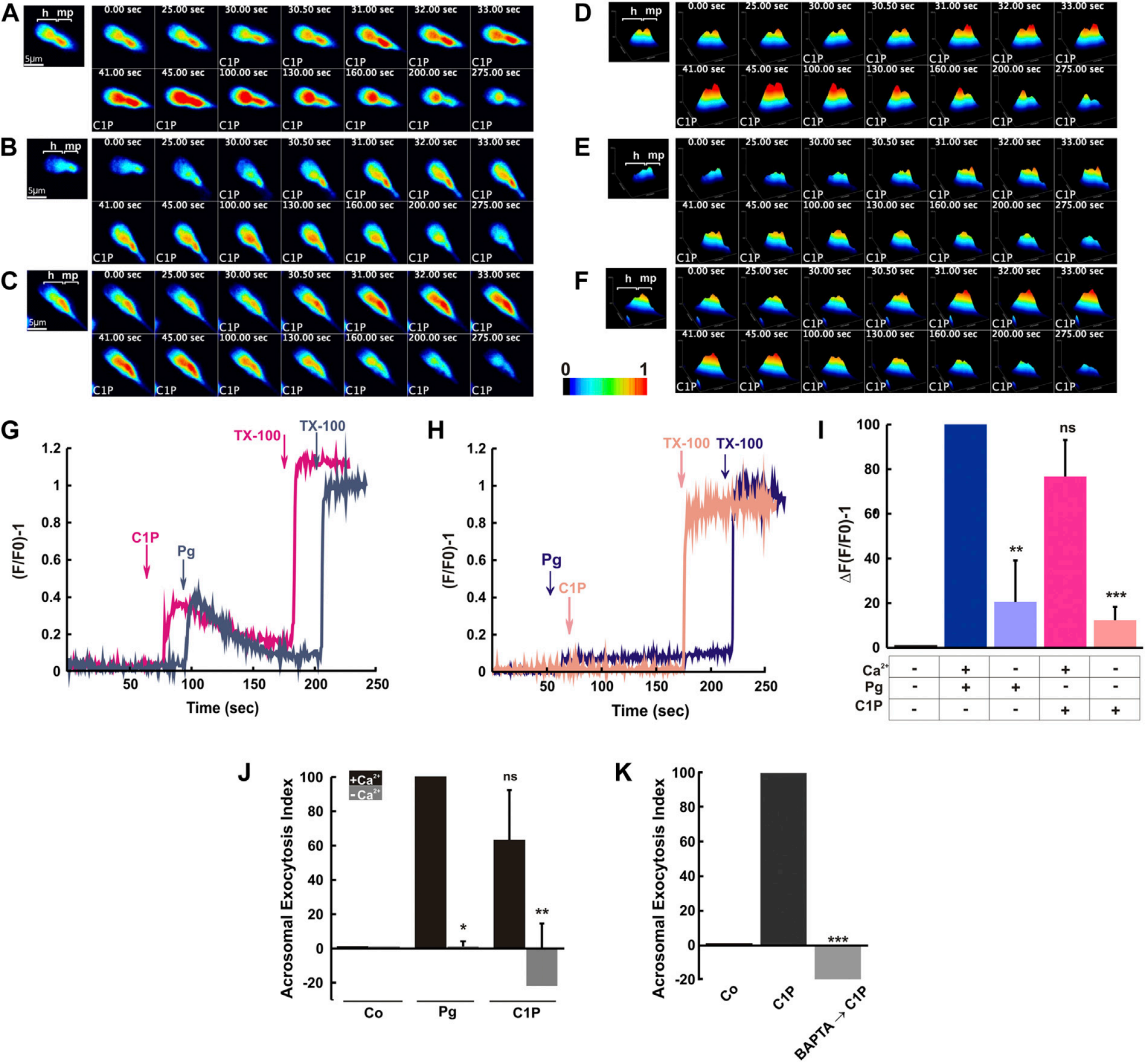
C1P-triggers extracellular calcium influx leading to sperm acrosomal exocytosis. Capacitated human sperm were loaded with Fluo-3 AM (2 μM) and suspended in HTF medium containing a final concentration of 2 mM CaCl_2_. The fluorescence intensity was visualized before and after C1P addition as described under “Materials and Methods”. **(A–C)** Sequence of fluorescence images from human sperm capacitated and loaded with Fluo-3AM showing changes in [Ca^2+^]i before (0 s) and after (25, 30, 30.5, 31, 32, 33, 41, 45, 100, 130, 160, 200, and 275 s) the application of 10 μM C1P. Shown are representative images of three individual human sperm. **(D–F)** Surface plots of the cells shown in panels A–C, also plots indicate the increase in fluorescence intensity in response to 10 μM C1P. Fluorescence is expressed as (F/F0) −1 *versus* time. Pseudocolor from black to red represents low to high [Ca^2+^]i, respectively. h: head, mp: midpiece. **(G)** Calcium sperm response to C1P in population. Human sperm were loaded with 2 μM Fluo-3 AM for 30 min at 37°C. At the indicated times (arrows) 10 μM C1P or 15 μM progesterone (Pg) were added. Maximal [Ca^2+^]i response was calibrated with 0.1% Triton X-100 (TX-100) at the end of the incubation period. Shown are traces representative of 6 experiments. The increase in fluorescence is expressed as (F/F0) −1 ((maximum fluorescence intensity/initial fluorescence) - 1) *versus* time in seconds. **(H)** Capacitated male gametes were suspended in the same medium including 0.75 mM EGTA to chelate the calcium leaving the HTF with a [Ca^2+^] ≤ 100 nM. Then, cells were loaded with 2 μM Fluo-3 AM for 30 min at 37°C. At the indicated times (arrows), 10 μM C1P or 15 μM progesterone (Pg) were added. Maximal [Ca^2+^]i response was calibrated with 0.1% Triton X-100 (TX-100) at the end of the incubation period. **(I)** Summary of Figures 2G,H. The data represent the mean ± S.E. of five experiments. **(J)** Acrosomal exocytosis was evaluated by FITC-PSA binding. We scored at least 300 cells per condition. Sperm batches incubated in normal calcium concentrations (black bars) and treated with 0.75 mM EGTA (gray bars) were challenged with 10 μM C1P or 15 μM progesterone (Pg). The data represent the mean ± S.E. of at least five independent experiments. The means of groups + Ca^2+^ and -Ca^2+^ were compared to Pg treatment (2 mM Ca^2+^) using Dunnett’s test and classified as non-significant (ns, *p* > 0.05) or significant (*, *p* ≤ 0.05; **, *p* ≤ 0.01). **(K)** Capacitated sperm were incubated with 5 mM BAPTA for 15 min and after that, C1P was added and further incubated for 15 min. Acrosomal exocytosis was evaluated by FITC-PSA binding scoring at least 300 cells per condition. The data represent the mean ± S.E. of four independent experiments. We used Dunnett’s test to compare the means of all groups against the Control condition and classified them as significant (***, *p* ≤ 0.001).

First, we suspended the live gametes in HTF medium containing a final concentration of 2 mM CaCl_2_. After that, we performed single-cell experiments by loading spermatozoa with Fluo-3 AM dye, a permeant Ca^2+^ indicator, and measuring Ca^2+^ changes in an inverted fluorescence microscope. Figure 2A–C displays sequential images of human sperm after 10 μM C1P addition. We showed the [Ca^2+^]i changes in three live single cells from 0 to 275 s. We noticed the cells presented different kinetic responses after the stimulus addition. Further, the cells recovered the basal calcium values indicating that the ion homeostasis has been restored. The kinetic of the calcium changes can be observed in the Supplementary Movies S1–S3. Figure 2D–F illustrated a surface plot of each cell. Summarizing, exogenous C1P provokes an [Ca^2+^]i rise in human sperm. Next, we analyzed calcium sperm response to C1P in the population. Sperm suspended in HTF and loaded with the calcium indicator were incubated with C1P or Pg. C1P caused a sperm [Ca^2+^]i augment followed by a gradual drop to almost resting levels (Figure 2G). This transient calcium increase is coincident with that induced by 15 μM Pg treatment (control).

To establish whether the C1P-induced [Ca^2+^]i increase was due to calcium influx from the extracellular medium or calcium mobilization from the internal reservoirs, we decided to measure [Ca^2+^]i in the sperm population in a free calcium media. For this purpose, we resuspended capacitated sperm in the same medium including 0.75 mM EGTA to chelate the calcium leaving the HTF with a [Ca^2+^] ≤ 100 nM as previously described in Vaquer et al. (2020) (Vaquer et al., 2020). Afterward, we loaded the male gametes with the permeant calcium probe. Under this extremely low extracellular [Ca^2+^] C1P did not cause an increase in sperm [Ca^2+^]i. The same result was obtained by adding Pg under the same conditions (Figure 2H). Different sperm batches achieved equivalent results that were depicted and compared in Figure 2I. These results indicate that C1P elicited human sperm transitory [Ca^2+^]i rise is due to extracellular calcium entry.

We evaluated the C1P effect on the AR in a calcium free media. As shown in Figure 2J, incubating the cells in HTF media with [Ca^2+^] ≤ 100 nM abolished the exocytotic effect of C1P and Pg. Pg-induced AR depends on calcium entry (Carlson et al., 2022), and the result was reproduced here in Figure 2J as a control. Further, sperm incubated in normal HTF (2 mM CaCl_2_) and then treated with 5 mM BAPTA for 15 min were not able to respond to the C1P stimulus. The incubation with the chelating agent for a short time was enough to inhibit the AR (Figure 2K).

The last results indicate that C1P induces Ca^2+^ influx from the extracellular media, which is necessary for the lipid-triggered exocytosis.

### C1P increases intracellular Ca^2+^ in human sperm through the activation of VOCCs, SOCCs, CatSper, RyR, and IP_3_-dependent calcium channels

**FIGURE 3:**
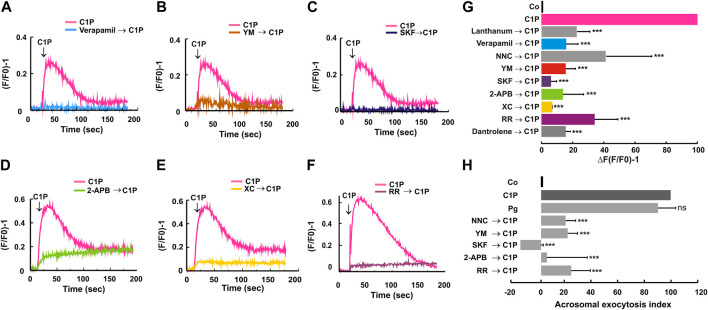
C1P-elicited intracellular Ca^2+^ increase relies on extracellular calcium influx and the ion release from intracellular stores. Capacitated human sperm recovered after swim-up were loaded with Fluo-3 AM (2 μM), and the fluorescence intensity was observed as described under “Materials and Methods”. Representative spatiotemporal [Ca^2+^]i changes and their corresponding traces are shown. The arrow indicates the addition of 10 μM C1P. When indicated, cells were previously incubated for 10 min at 37°C with: **(A)** 100 μM Verapamil, **(B)** 1 μM YM-58483 (YM), **(C)** 50 μM SKF-96365 (SKF), **(D)** 100 μM 2-APB, **(E)** 1 μM Xestospongin C (XC), **(F)** 20 nM ruthenium red (RR). **(G)**, ΔF(F/F0) − 1 is the average of the changes in fluorescence of each condition measured. Here, we represented in addition results obtained after the treatment with 200 μM Lanthanum, 1 μM NNC, and 100 μM Dantrolene. Bars represent the mean ± S.E of five independent experiments. Different conditions were compared using Dunnett’s test and C1P as a control, and differences were classified as significant (***, *p* ≤ 0.001). **(H)** To determine if calcium channels inhibitors affect the AR we incubated capacitated human sperm without any treatment (control, Co) or treated when indicated with, 1 μM NNC, 1 μM YM, 50 μM SKF, 100 μM 2-APB, or 20 nM RR for 15 min at 37°C. When specified, acrosomal exocytosis was initiated by adding 10 μM C1P and the incubation continued for an additional 15 min. We used 15 μM Pg as a positive control of the experiment. Sperm were then fixed, and acrosomal exocytosis was measured. The data represent the mean ± S.E. from 3 to 5 independent experiments. Dunnett’s test was used to compare the means of all groups against the C1P-stimulated condition in the absence of inhibitors and classified as non-significant (ns, *p* > 0.05) or significant (***, *p* ≤ 0.001).

The classical calcium channels pathway requires different calcium waves produced by the sequential activation of these channels. In short, an exocytotic stimulus provokes VOCCs opening that generates a transient calcium increase inducing reservoirs (acrosome and RNE) emptying. The voiding of the stores causes a sustained calcium influx through SOCCs. We decided to analyze which calcium channel/s are implicated in the C1P-triggered mechanism. To test this premise, we incubated Fluo-3 AM-loaded sperm in HTF medium containing 2 mM CaCl_2_. Then, we added specific inhibitors of calcium channels and measured, in the sperm population, the variations in [Ca^2+^]i after cells were challenged with 10 μM C1P. First, we treated sperm with 200 μM lanthanum, which is a general calcium channel blocker. C1P-induced calcium rise was sensitive to lanthanum suggesting the requirement of plasma membrane calcium channels (Figure 3G).

To block L-type high voltage-activated Ca^2+^ currents we used 100 μM verapamil. The arylalkylamine treatment abolished completely the C1P-induced calcium increase (Figures 3A,G). It is expected that an early transient calcium rise through VOCCs, activates the emptying of calcium stores through IP_3_R and this calcium efflux is mandatory for the AR. Therefore, we incubated sperm with IP_3_-sensitive calcium channel blockers before C1P addition. Both 2-APB and Xestospongin C (XC) inhibited significantly the C1P-triggered calcium increase (Figures 3D, E, G). The RNE has been described as a calcium reservoir in mammalian sperm (Harper and Publicover, 2005; Suarez, 2008). Both IP_3_R and RyR are present in its membrane (Reviewed in Darszon et al., 2011 (Darszon et al., 2011)). Since we described a function for these RyRs in the ceramide-elicited acrosome exocytosis we asked if they are required for calcium signal induced by C1P. We incubated the cells with 20 nM ruthenium red (RR) or 100 μM dantrolene to block the RyR function. The treatments prevented the sphingolipid-induced calcium augment, indicating that the RNE is an important store involved in C1P-elicited calcium signaling (Figures 3F,G). To elucidate if plasma membrane calcium channels like SOCCs, are required for the C1P-stimulated calcium rise we resorted to specific blockers: SKF-96365 (Kachoei et al., 2006; Trevino et al., 2006) and YM-58483 (Yoshino et al., 2007). Both inhibitors blocked calcium increment elicited by C1P (Figures 3B,C,G). CatSper has been proposed as the plasma membrane Ca^2+^ channel responsible for the initial Ca^2+^ transient current (Kirichok et al., 2006). NNC was reported as a CatSper blocker (Lishko et al., 2011; Strunker et al., 2011). NNC inhibited significantly C1P-triggered [Ca^2+^]i augment (Figure 3G). These last results suggest that even though the extracellular calcium entry is necessary for C1P-induced [Ca^2+^]i augment both plasma membrane and store channels participate in calcium rise and the acrosomal exocytosis.

Since not all the calcium augments observed lead to the sperm AR, we measured exocytosis after inhibiting the aforementioned channels under the same conditions. The use of these channel blockers significantly inhibited the C1P-provoked exocytosis (Figure 3H).

### CERK is present and catalyzes C1P synthesis in response to calcium increase in human spermatozoa

Previously, our laboratory demonstrated a crucial role for S1P as a sperm acrosomal exocytosis inducer. Spermatozoa exocytic stimuli elicit S1P synthesis, which can reach the extracellular medium and bind to Gi-coupled receptor/s triggering a signaling cascade that drives the AR (Suhaiman et al., 2010). Next, we described that ceramide triggers the acrosomal exocytosis by inducing calcium mobilization from internal stores and external calcium entry conducting, finally to sperm AR (Vaquer et al., 2020). In that last publication, we first hypothesized that the ceramide increase could be inducing S1P synthesis given the presence in sperm of the sphingolipid metabolism enzymes required (Suhaiman et al., 2010). However, against our prediction, the sphingosine kinase inhibitor does not hinder the ceramide-stimulated AR. This demonstrates that ceramide elicits exocytosis in an S1P-independent way (Vaquer et al., 2020). Moreover, we described the ceramide pathway that differs at the early stages from that induced by S1P.

**FIGURE 4:**
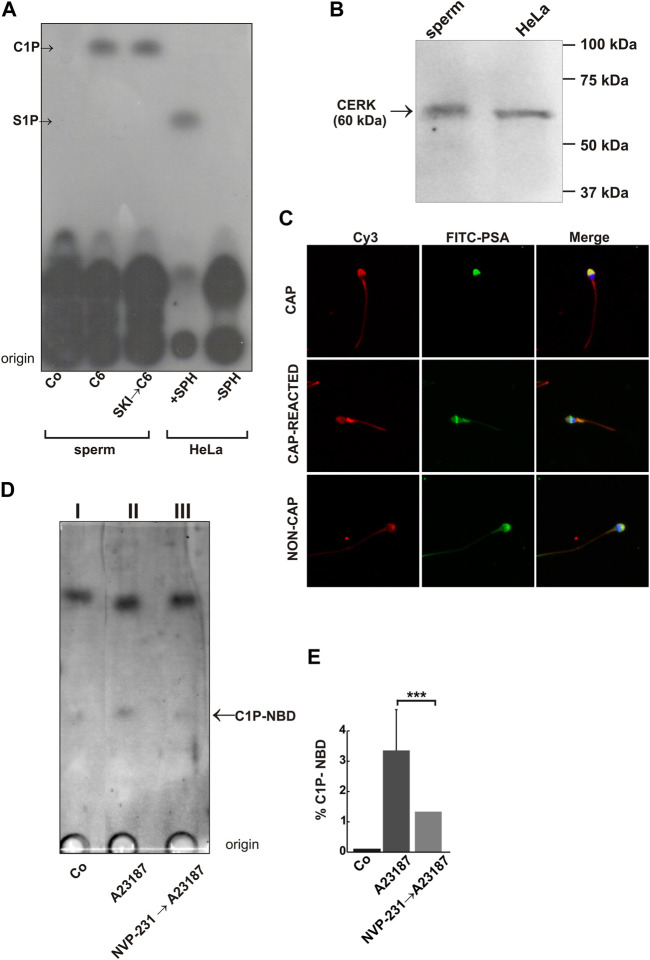
C6-Ceramide induces C1P synthesis in human spermatozoa. **(A)** After swim-up in HTF at 37°C, 5% CO_2_, sperm were incubated under capacitating conditions for 2 h, and permeabilized with streptolysin O as described in our previous publications (Lopez et al., 2012; Pelletan et al., 2015). When indicated, we incubated permeabilized sperm in SK1 buffer containing 1 μM SKI (specific Sphingosine Kinase Inhibitor) for 15 min at 37°C. We further incubated the samples for 15 min at 37°C with 50 μM fatty acid free bovine serum albumin-sphingosine, 1 μL of [γ-^32^P]ATP in the absence of any stimulus (Control, Co) or 10 μM C6-Cer (C6, SKI→C6). HeLa cells (positive control, incubated with sphingosine; +SPH, and negative control with no sphingosine added; -SPH) were prepared as described under “Materials and Methods”. Thin-layer chromatograms were developed in 1-butanol/methanol/acetic acid/water (8:2:1:2, v/v) and observed by autoradiography. The figure is representative of three experiments. **(B)** CerK is present and catalyzes C1P synthesis in response to calcium increase in human spermatozoa. Sperm proteins were extracted in Laemmli sample buffer (10 × 10^6^ cells) and analyzed by Western blot with a rabbit polyclonal anti-CERK antibody (sperm). HeLa extract (HeLa) was used as a control. **(C)** Capacitated (CAP) and non-capacitated (NON-CAP) human sperm were fixed and double-stained with an anti-CERK antibody followed by an anti-rabbit Cy3 (red: Cy3) and FITC-PSA to differentiate between reacted and intact sperm (green: FITC-PSA). The middle panel shows a capacitated cell that in response to the Pg stimuli lost its acrosome (CAP-REACTED) and Cy3 staining remains diffuse. Merged images are shown (stained sperm nuclei are visualized in blue, Merge). **(D)** Capacitated sperm were loaded with a fluorescent ceramide (4-nitrobenzo-2-oxa-1,3-diazole–labeled C12-ceramide, NBD-ceramide). After that, cells were incubated or not (Co, basal activity) with a calcium ionophore, A23187, to increase the [Ca^2+^]i. One batch of ionophore-treated cells was previously incubated with the CERK inhibitor, NVP-231 (adamantane-1-carboxylic acid (2-benzoylamino-benzothiazol-6-yl)amide) 200 nM. Before resolving ceramide metabolites by TLC, we separated them according to their lipophilicity as described in “Materials and Methods”. **(E)**. Quantification of spot intensity of three thin-layer chromatograms corresponding to C1P-NBD. A significant increase of C1P-NBD was assessed by *t*-test for single group mean significant (***, *p* ≤ 0.001).

Here, we decided to measure if ceramide can induce the synthesis of a phosphorylated bioactive sphingolipid once added to sperm. Then, spermatozoa were incubated or not with 10 μM ceramide (C6) in the presence of [γ-^32^P] ATP. The ceramide treatment did not induce S1P synthesis in human sperm. HeLa cells incubated with sphingosine proved the Rf of S1P (Figure 4A). However, here we identified a spot in sperm samples incubated with ceramide consistent with the Rf of C1P. The sperm incubation with the Sphingosine Kinase Inhibitor (SKI) before the C6 addition did not affect the C1P synthesis (Figure 4A, C6, and SKI →C6). These results demonstrate that an increase of ceramide in sperm provokes an acute C1P rise. Furthermore, they imply the existence of an active CERK in human sperm. To determine if this kinase was present in spermatozoa we performed Western blot assays. We utilized a specific polyclonal antibody (Abcam) to reveal the presence of CERK in sperm extracts. Figure 4B (whole sperm extract, sperm) showed a single band of an apparent MW of ∼60 kDa equivalent to the MW described for human CERK, and an identical band in a homogenate of HeLa cells (control, Figure 4B, HeLa lane). To analyze the CERK localization we used indirect immunofluorescence. To differentiate between intact and reacted spermatozoa we performed double labeling with FITC-PSA (Figure 4C, FITC-PSA, and Cy3). Both, capacitated and non-capacitated (CAP, NON-CAP) sperm showed strong labeling in the acrosomal region and the midpiece of the flagellum. This pattern was present in 82% of the non-reacted cells (n = 4 samples). When capacitated samples where stimulated with Pg (CAP-REACTED), the cells lost their acrosomes and the acrosomal region labeling is replaced by a diffuse staining pattern.

CERK catalyzes the phosphorylation of ceramide to synthesize the bioactive sphingolipid C1P. The kinase contains a Ca^2+^/calmodulin (Ca^2+^/CaM) binding motif (Rhoads and Friedberg, 1997), which is required for C1P production in response to [Ca^2+^]i increase (Mitsutake and Igarashi, 2005; Hinkovska-Galcheva and Shayman, 2010). Hence, we decided to evaluate if the sperm CERK is active, upregulated by calcium, and inhibited by a specific CERK blocker. For this purpose, we made use of the thin-layer chromatography (TLC) method we published recently for sperm sphingolipid metabolites (Vaquer et al., 2020). First, we loaded sperm with a fluorescent ceramide (4-nitrobenzo-2-oxa-1,3-diazole–labeled C12-ceramide, NBD-ceramide). After that, cells were incubated or not (Co, basal activity) with a calcium ionophore, A23187, to increase the [Ca^2+^]i. One batch of ionophore-treated cells was previously incubated with the CERK inhibitor, NVP-231. A potent, specific, and reversible CERK inhibitor that competitively inhibits ceramide binding to CERK. It abrogates phosphorylation of ceramide, resulting in decreased endogenous C1P levels (Graf et al., 2008). Before resolving ceramide metabolites by TLC, we separated them according to their lipophilicity. Then, we measured C1P levels in the aqueous phase. Figures 4D,E showed a low basal activity of the enzyme (Co) in human sperm. The calcium ionophore, A23187, increased significantly the kinase activity, which was almost abolished in the presence of NVP-231. Thus, calcium is a CERK activator in human sperm AR and NVP inhibits the kinase activity allowing us to use this permeant inhibitor as a tool for exocytosis experiments.

### Ceramide induces sperm acrosomal exocytosis, partly, due to C1P synthesis

**FIGURE 5:**
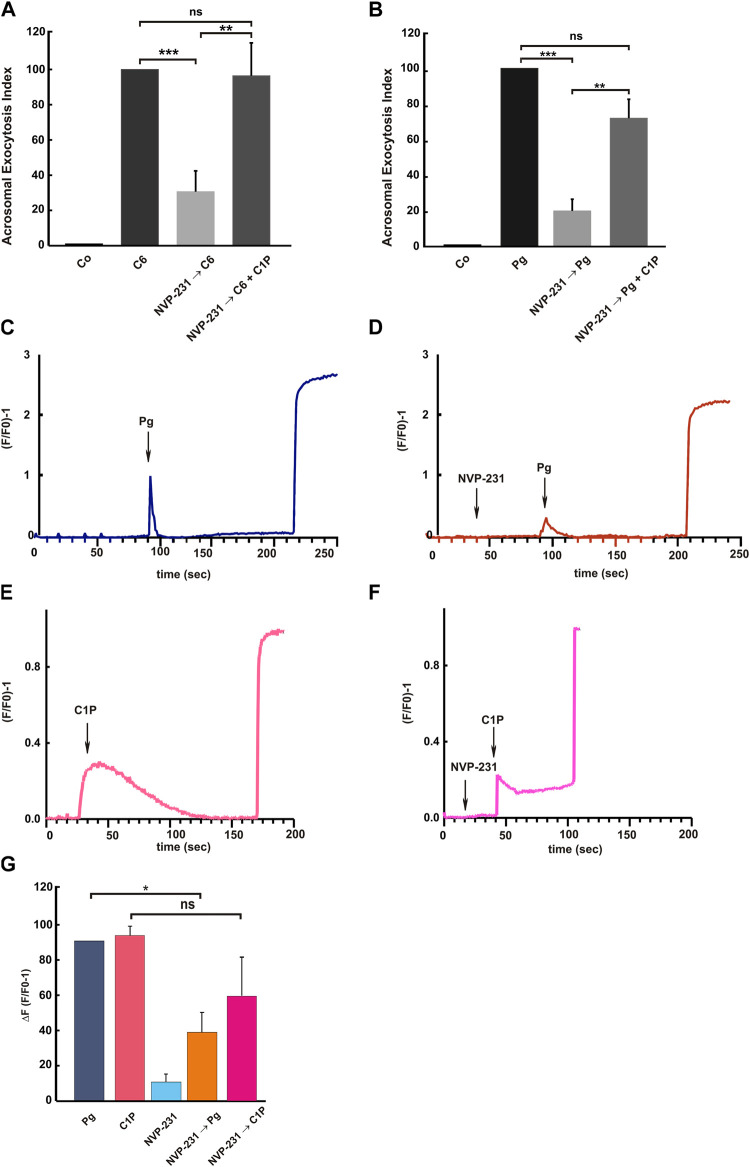
Ceramide and Progesterone require C1P to trigger acrosome exocytosis. After swim-up in HTF (5 mg/ml BSA) at 37°C, 5% CO_2_, sperm were incubated for an additional 2 h under capacitating conditions. **(A)** Capacitated sperm were treated with 200 nM NVP-231 for 15 min at 37 °C in 5% CO_2_ and further incubated with 10 μM C6-Cer (NVP-231→C6) or with 10 μM C6-Cer plus 10 μM C1P (NVP-231→C6+C1P). C6-Cer (C6) was used as a positive control **(B)** We incubated capacitated spermatozoa with 200 nM NVP-231 for 15 min at 37°C in 5% CO_2_ then we treated the cells for an additional 15 min with 15 μM Pg (NVP-231→Pg, Pg) or with 15 μM Pg plus 10 μM C1P (NVP-231→Pg + C1P). Sperm were fixed and acrosomal exocytosis was evaluated by FITC-PSA binding with at least 300 cells per condition scored. Tukey’s test was used to compare mean groups of treatments and classified as non-significant (ns, *p* > 0.05) or significant (**, *p* < 0.01 or ***, *p* < 0.001). **(C–F)** Capacitated human sperm were loaded with 2 μM Fluo-3 AM for 30 min at 37°C. At the indicated times (arrows) 15 μM Pg was added. Maximal [Ca^2+^]i response was calibrated with 0.1% Triton X-100 (TX-100) at the end of the incubation period. When indicated, 200 nM NVP-231 was added before Pg or C1P addition. Shown are traces representative of 4 experiments. The increase in fluorescence is expressed as (F/F0) −1 ((maximum fluorescence intensity/initial fluorescence) - 1) *versus* time in seconds. **(G)** ΔF(F/F0) − 1 is the average of the changes in fluorescence of each condition measured. Bars represent the mean ± S.E of 4 independent experiments. Different conditions were compared using Tukey’s test and the differences were classified as significant (*, *p* < 0.05; NVP-231→Pg, Pg), or as non-significant (ns, *p >* 0.05; NVP-231→C1P, C1P).

A few years ago, we unveiled the exocytic pathway triggered by ceramide. The sphingolipid induces a multicomponent calcium rise driving exocytosis. The calcium channels described to be regulated by ceramide constitute a calcium signaling partly similar to that induced by C1P. Here we demonstrated that ceramide induces C1P synthesis in human sperm. Strikingly, C1P elicited exocytosis by itself. Then, we asked whether ceramide-accomplished exocytosis is due to C1P synthesis. To answer this question, we incubated capacitated sperm with NVP-231, the CERK inhibitor, before adding the ceramide stimulus. We hypothesized that if ceramide was exerting its effect through C1P the exocytosis will be completely abolished. As shown in Figure 5A, the NVP-231, inhibited significantly ceramide-triggered exocytosis. Even so, 36% of the relative AR remained. C1P completely rescued the inhibition of ceramide-triggered exocytosis caused by the CERK blocker, evincing the specificity of the NVP-231.

### Progesterone requires C1P to trigger acrosome exocytosis

There are two well-known physiological inducers of the AR: Pg and zona pellucida (ZP) glycoproteins (Aldana et al., 2021). However, the complete network of molecules involved in both pathways is largely unknown. In particular, the function of lipids has been relegated to a secondary role in a signaling cascade dominated by proteins. To determine the physiological importance of C1P in the progesterone pathway, we stimulated the AR with Pg in the presence of the CERK inhibitor. NVP-231 inhibited significantly (80%) the Pg-elicited exocytosis. C1P reversed the inhibitory effect. This result highlight the role of the bioactive sphingolipid C1P as a molecule implied in the acrosomal exocytosis physiology.

Trying to understand which is the role of the C1P in the Pg pathway, we inquired if the CERK activity was necessary for the Pg-induced calcium increase. Then, we resort to calcium measurements in cell population. We loaded capacitated sperm with the calcium indicator Fluo3-AM. Next, we added the CERK inhibitor NVP-231, followed by the Pg stimulus. NVP decreased the calcium sperm response to Pg addition, causing a significantly drop in the (F/F0) – 1 (Figure 5D) compared to the transient calcium increase induced by Pg (Figure 5C). As a specificity control we added 10 μM C1P after NVP-231 treatment. C1P induced a calcium augment, which was not significantly different to that induced by incubating the cells with NVP before C1P addition (Figures 5E,F). The ΔF of all experimental conditions were compared in Figure 5G. To sum up, the calcium rise induced by Pg, partially, relies on CERK activity and consequently on C1P synthesis.

## Discussion

Whilst protein complexes implicated in exocytosis are relatively well characterized, few data are showing directly the mechanistic role of lipids during membrane fusion.

Further, scarce information exists about the biological significance of different lipids in human sperm AR. Given that the spermatozoa are conveniently fitted to study this issue, our laboratory has elucidated the signaling cascades accomplished by different sphingolipids during human sperm acrosomal exocytosis establishing a dynamic protein-membrane interface (Suhaiman et al., 2010; Belmonte & Suhaiman, 2012; Vaquer et al., 2020). Recently, we described that ceramide increases intracellular calcium by activating SOCCs, CatSper, and RyRs triggering human sperm AR (Vaquer et al., 2020). Ceramide is considered a main hub in the *de novo* sphingolipids synthesis generating a large variety of molecules. Given this, we focused on the bioactive metabolites synthesized. As previously demonstrated, ceramide, a precursor of S1P, does not require S1P presence to activate exocytosis. Herein, we confirmed that ceramide addition to sperm does not induce acute S1P synthesis but the small bioactive lipid C1P. The specific aim of this research was to analyze whether C1P is involved in sperm AR. To the best of these authors’ knowledge, the implication of C1P in sperm AR has not been investigated, nor has its physiological importance. Here, for the first time, we evaluated this issue using human sperm allowing an extrapolation and transference to the clinical interests.

Given that we added exogenous C1P to the extracellular media, which is not a membrane-permeant molecule, and observed its ability to induce the AR we conclude that our results support the idea of an extracellular role of C1P. Since C1P acts intra and extracellularly in other cells, we corroborated that the sphingolipid did not exert any effect in controlled plasma membrane permeabilized sperm (Figure 1). It is important to highlight that, C1P can be found in the intracellular and extracellular media. The sphingolipid is synthesized intracellularly in somatic cells. A calcium-dependent CERK catalyzes ceramide phosphorylation. The sphingolipid produced intracellularly can elicit numerous biological effects. On the other hand, C1P can be transported through the plasma membrane to the extracellular environment by a C1P transfer protein (CPTP) (Presa et al., 2020). C1P is present in plasma at concentrations ranging from 0.5 to 1.5 μM. Extracellular C1P stimulates cell migration, glucose uptake, and adipogenesis inhibition through a Gi protein-coupled receptor-mediated mechanism. However, even if a putative C1P receptor has been in part characterized, receptor/s have not yet been isolated nor cloned (Arana et al., 2013; Ouro et al., 2013). In intact human sperm, 0.1 μM C1P evoked the maximal exocytic response indicating that a high-affinity receptor is mediating C1P action. Our results support the idea that C1P interacts with a C1P receptor in the sperm plasma membrane allowing the bioactive sphingolipid to trigger a signaling mechanism leading to AR.

In addition, the capacitation is a requirement for C1P-induced acrosome exocytosis, suggesting a physiological behavior of the lipid. We assessed by TEM that C1P provokes the physiological activation of the AR at the ultrastructural level. The results obtained are comparable to that induced by Pg, which have been demonstrated to be similar to that elicited by the calcium ionophore, A23187 (Zanetti & Mayorga, 2009; Sosa et al., 2015; Sosa et al., 2016). Further, the necessity of a functional VAMP2 to engage the fusion of the outer acrosomal membrane with the plasma membrane during C1P-elicited exocytosis rule out the possibility of an artifactual effect of the lipid on exocytosis. These results discard the probability of acrosome loss due to C1P-induced membrane destabilization.

C1P has been proposed to stimulate exocytosis by rising the fusion ability of the synaptic vesicle membranes. Both C1P phosphatase and CERK, are associated with these organelles (Bajjalieh et al., 1989; Sugiura et al., 2002). Liposomes shifted the rate and extent of their calcium-reliant fusion when C1P is added (Hinkovska-Galcheva et al., 1998). Hori et al. (2020) demonstrated that in PC12 cells the CERK/C1P pathway plays a pivotal role in stimulating noradrenaline release. The sphingolipid seems to be directly stimulating the exocytic machinery (Hori et al., 2020). Even though the molecular mechanisms activated by this lipid in exocytosis have not been elucidated yet. However, given that regulated exocytosis requires a fine-tuning of [Ca^2+^]i, how the activation of the CERK/C1P pathway regulates this ion leading to exocytosis, is an unresolved topic. This complex function requires multiple channels, transporters, and pumps. Our calcium measurement results performed in single-cell and population (Figure 2), lead us to conclude that C1P induced calcium entry from the extracellular media through VOC and SOC channels and CatSper. However, the calcium release from internal stores *via* RyR and IP_3_R-dependent calcium channels was essential for C1P-triggered exocytosis to proceed. We hypothesize that exogenous C1P interacts with a Gi-coupled receptor activating a transient VOCCs opening and triggering the canonical exocytotic pathway described for sperm (Suhaiman et al., 2010; Darszon et al., 2011). We aim to emphasize that the calcium kinetics during the first 200 s is coincident with that activated by progesterone, the physiological AR inducer rising the physiological importance of the findings.

We described previously the exocytic pathway triggered by endogenous and exogenous added ceramide, the immediate precursor of C1P, to the male gamete (Vaquer et al., 2020). Given that, both, ceramide and its phosphorylated product, trigger the AR is imperative to compare the effect of the sphingolipids on calcium channels. Ceramide was able to raise [Ca^2+^]i even in the absence of extracellular calcium ([Ca^2+^] ≤ 100 nM) due to its ability to mobilize the ion from internal stores. We introduced evidence demonstrating that after ceramide treatment, RyRs are activated. Notwithstanding, calcium efflux from internal reservoirs did not result sufficient to trigger the AR. Calcium entry from SOCCs and CatSper were mandatory for ceramide-elicited AR, as well as, a late calcium efflux from IP_3_-dependent calcium channels. Further, ceramide-evoked exocytosis does not require the VOCC’s activation. However, C1P, the metabolite synthesized 10 min after ceramide addition to sperm (Figure 4), provoked an [Ca^2+^]i increase that relies on different calcium channels that ceramide although needed extracellular calcium entry and intracellular stores calcium release. Even though, both lipids share some channels. We thought, that probably ceramide can initiate the pathway, increasing the [Ca^2+^]i which activates the CERK, generating C1P. Our hypothesis is that ceramide and C1P are in the same pathway leading to acrosome exocytosis. Probably, both sphingolipids are inducing temporally different calcium waves.

Our results are similar to those published by Colina et al., 2005 (Colina et al., 2005) where C1P increases [Ca^2+^]i in Jurkat T Cells. C1P causes an IP_3_ rise releasing calcium from the ER and provoking the opening of SOCCs at the plasma membrane. Hogback et al., 2003 (Hogback et al., 2003) described that in thyroid FRTL-5 cells, C1P evokes a pertussis toxin-sensitive G protein-mediated mechanism that activates a PLC and the sphingosine kinase (SK), leading to S1P synthesis. This result caught our attention because our group outlined the S1P pathway during acrosomal exocytosis (Suhaiman et al., 2010). In GH4C1 rat pituitary cells, C1P elicited Ca^2+^ influx from the media through VOC channels (Tornquist et al., 2004). Thus, C1P is a molecule able to induce calcium increase in different somatic cells using diverse mechanisms and probably by activating a receptor. Some of these molecular mechanisms are shared with those described for the male gamete in this study.

hCERK is a protein of 537 amino acids. It contains a catalytic domain with a high degree of analogy to the glycerol kinase catalytic region. The enzyme carries a Ca^2+^/calmodulin binding motif which regulates its activity and consequently, the C1P synthesis when [Ca^2+^]i increases (Mitsutake et al., 2004; Mitsutake and Igarashi, 2005). In this work, we demonstrated for the first time the presence of the CERK in the human male gamete, a protein with a MW of 60 kDa. The MW is coincident with that shown in somatic cells described in the literature (Sugiura et al., 2002) and HeLa cells (Figure 4). Moreover, Wang et al. (2013) reported the presence of the CERK in the human sperm proteome (Wang et al., 2013) supporting our findings. Concerning the localization, the kinase resides in several somatic cell compartments, e.g., the nucleus, the cytosol, and the plasma membrane. However, its main location is the Golgi apparatus. It has been described as a cytosolic enzyme that migrates to the membranes when active in somatic cells (Presa et al., 2020). The spermatozoa are polarized and very compartmentalized cells so, the enzyme is distributed in defined zones. At least, its presence in the acrosomal region matches its exocytic function, and this particular staining is lost after the AR. Remarkably, we found that the CERK activity is upregulated by calcium during human sperm AR and blocked by a specific CERK inhibitor. The kinase has been involved in the exocytosis of numerous cells. The CERK inhibition abolishes mast cells degranulation (Kim et al., 2005) and histamine and PGD2 release (Hewson et al., 2011). In addition, the CERK/C1P pathway plays a stimulatory role in the noradrenaline release in PC12 cells (Hori et al., 2020). Calmodulin directly interacts with CERK and is involved in the regulation of exocytosis, including acrosomal exocytosis (Lopez-Gonzalez et al., 2001; Yunes et al., 2002; Xue et al., 2021). On the other hand, CERK binds to a crucial lipid for sperm acrosomal exocytosis like PIP_2_ (Lopez et al., 2012; Pelletan et al., 2015), we believe that fits the lipid pathway we have described for sperm AR. In addition, CERK binding could be regulating the availability of the phospholipid for the IP_3_ and DAG synthesis thanks to PLC activation. Taking into account, that Ca^2+^ activates CERK during the AR and that C1P activates sphingosine kinase (SK1) (Nishino et al., 2019), we cannot discard that C1P could be inducing SK1 activation and consequently, S1P synthesis, which triggers the pathway we described for the AR (Suhaiman et al., 2010).

Both calcium and ceramide provoked C1P synthesis (Figure 4). Ceramide elicits the AR activating multiple calcium channels but here, we evinced that the sphingolipid requires the C1P synthesis to exert its full action (Figure 5). Moreover, the inhibition of the CERK restrained significantly Pg-induced AR and the Pg-elicited [Ca^2+^]i calcium augment. Consequently, Pg physiological activity in human sperm relies on ceramide (Vaquer et al., 2020) and C1P, however, is independent of S1P (Suhaiman et al., 2010). It is important to remark that, during exocytosis, there is an increase in intracellular C1P, nevertheless, the phosphosphingolipid only causes a biological effect when added to the extracellular media.

**FIGURE 6:**
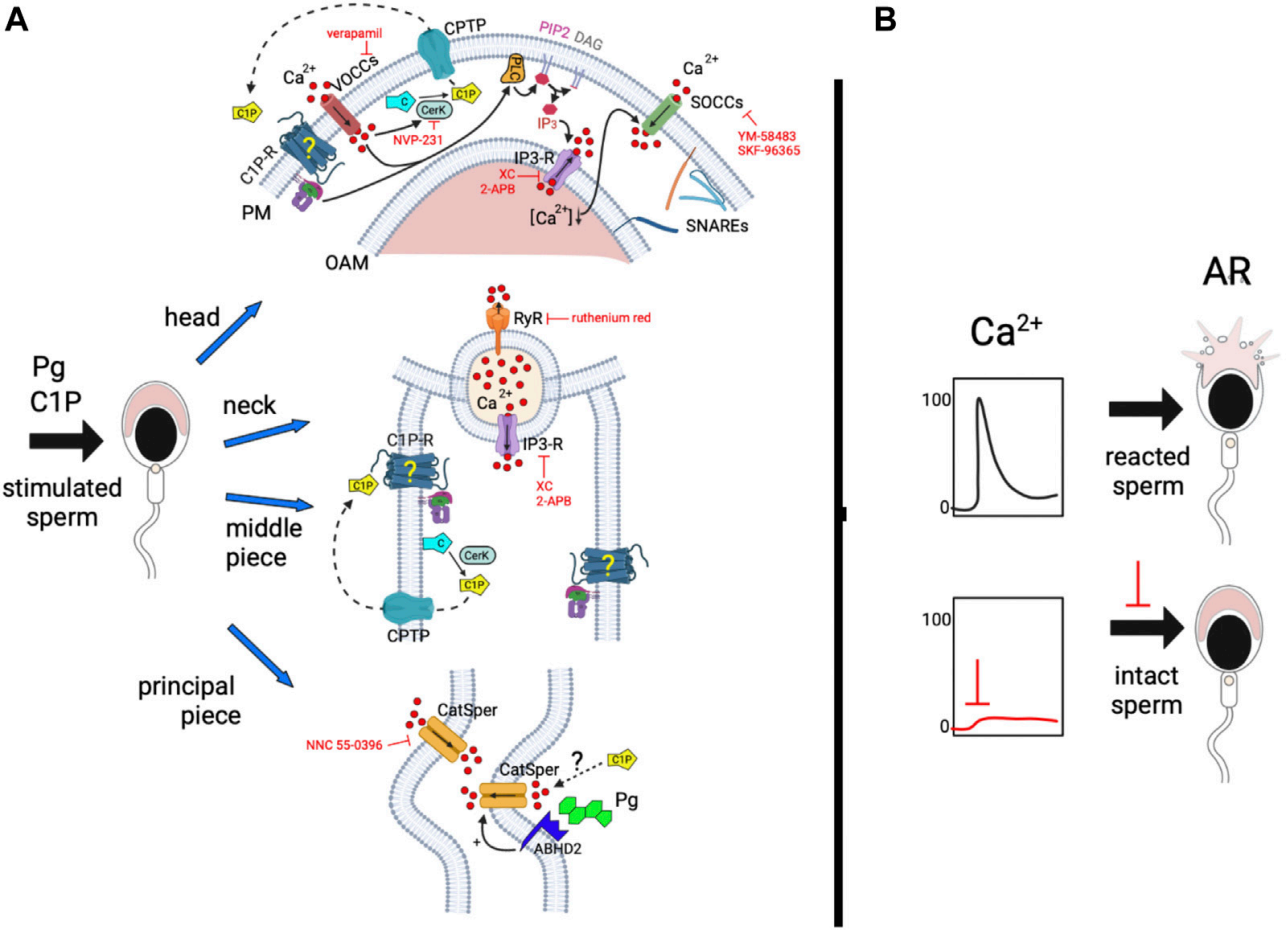
Scheme of the working model hypothesized for C1P/CERK pathway in acrosome exocytosis. **(A)** We propose that C1P interacts with a Gi-coupled receptor (C1P-R) activating VOC channels and a heterotrimeric Gi-protein. The transient calcium augment could be activating the CERK through the Ca^2+^/calmodulin-binding motif. The kinase phosphorylates ceramide to C1P, which can be transported through the plasma membrane to the extracellular environment by a C1P transfer protein (CPTP). Once outside, it can interact with its receptor amplifying the signaling pathway. Calcium increase and the heterotrimeric Gi-protein switch on a PLC which hydrolyzes phosphatidylinositol 4,5-bisphosphate (PIP_2_) producing DAG and IP_3_. The last one binds to IP_3_-sensitive calcium channels, present on the outer acrosomal and redundant nuclear envelope (RNE) membranes, inducing calcium efflux from the reservoirs. The voiding of the stores triggers the opening of SOCCs at the plasma membrane allowing a sustained calcium increase. At the same time, C1P, due to the first calcium wave can be causing the opening of ryanodine sensitive-calcium channels (RyR) present in the RNE. CatSper is a multimeric voltage-activated channel, negatively regulated by 2-arachidonoylglycerol (2-AG), which is hydrolyzed by the lipid α/β hydrolase domain-containing protein 2 when Pg binds its receptor. Given that C1P-induced AR requires CatSper activity, we suppose that the sphingolipid can set off CatSper *via* an unknown, alternative mechanism or by using the same machinery utilized by Pg. The last possibility makes sense due to the CERK activity requirement for Pg-triggered exocytosis. On the other hand, C1P drives SNARE (SNAP receptor) complex assembly during sperm. **(B)** C1P-elicited exocytosis is a complex mechanism that demands the participation of multiple calcium channels to reach a sustained [Ca^2+^]i necessary for the efficient signaling conducting human sperm AR.

In light of these new and previous results, we built a working model shown in Figure 6. We hypothesize that C1P binds to a putative Gi-coupled receptor, and induces heterotrimeric Gi-protein and VOCCs activity driving the PLC activation. PLC hydrolyzes PIP_2_ producing IP_3_ and DAG. IP_3_ binds to IP_3_-sensitive calcium channels present in the sperm acrosome and RNE membranes, releasing calcium from these stores and, stimulating SOCCs’ opening. Besides, cytosolic calcium increase could activate RyR at the sperm neck and, consequently, calcium efflux from the RNE. The activation of CatSper by C1P, a voltage-reliant, and pH-sensitive calcium channel, made us think about the molecular mechanism triggered by the sphingolipid. Evidence in the literature shows that ceramide 1-(2-cyanoethyl) phosphate (C1CP), a molecule generated during the final phase of C1P synthesis, induces ion changes in different cells. In thyroid FRTL-5 cells, patch-clamp assays demonstrated that C1CP hyperpolarized the membrane potential of the cells (Tornquist et al., 2002; Tornquist et al., 2004). We could hypothesize that C1P could be activating CatSper by modulating intracellular voltage. Even that, we cannot discard a direct effect of the phosphosphingolipid on the channel. In order to determine whether C1P/CERK signaling has a direct impact on CatSper currents in the Pg pathway, it would be required to conduct electrophysiological recordings.

It is easy to have straightforward thinking about ceramide and C1P connection given that the former is the immediate precursor of the phosphorylated sphingolipid and, we showed that ceramide acute increase produces C1P synthesis. In general, our results support the idea that they can act sequentially in the same pathway.

How would it be possible that Pg necessitates C1P synthesis to carry on exocytosis and [Ca^2+^]i increase? We propose that Pg, present in the human tubal fluid, induces a calcium increase that stimulates human sperm CERK to generate C1P. It is well known that Pg once coupled to its membrane receptor, activates an α/β hydrolase domain-containing protein 2 (ABHD2) (Miller et al., 2016). ABHD proteins play a crucial role in lipid metabolism, metabolic diseases, and lipid signaling. ABHD2 is a lipid hydrolase that acts depleting endocannabinoid 2-arachidonoylglycerol (2-AG), a natural inhibitor of CatSper, releases glycerol, arachidonic acid (AA), and increases [Ca^2+^]i. The [Ca^2+^]i increase elicited by Pg can activate the CERK in the male gamete generating C1P, which could be delivered to the extracellular media through the C1P transfer protein (CPTP). Once outside, C1P may bind to a putative C1P receptor sparking an exocytotic signaling cascade. Our research evinces that C1P is not only a booster producing a positive feedback loop for the AR. The inhibition of the CERK almost abolishes the Pg-induced acrosomal exocytosis and calcium augment pointing out the requirement of the phosphorylated sphingolipid during the physiological road to fertilization.

Recently, Shan et al. (2020) demonstrated that a specific hydrolase form (ABHD16B) is associated with bull infertility in Holstein cattle (Shan et al., 2020). Sperm lipidomics proved that the absence of ABHD16B influenced the content of ceramide, phosphatidylcholine, sphingomyelin, and diacylglycerol. The ABHD16B impaired function provoked an alteration of the sperm membrane composition. Therefore, we believe that the Pg signaling involves more lipids than we had thought until now. This follows our results published previously (Vaquer et al., 2020) and supports the role of ceramide and C1P in the Pg pathway.

Considering that, C1P is present at low concentrations in different biological fluids is a stable, slowly metabolized molecule, and possesses a long half-life (Gomez-Munoz et al., 1995; Gangoiti et al., 2012) we cannot discard that extracellular C1P in the female genital tract could trigger acrosomal exocytosis when the gamete transits near the oocyte. Further, Dr. Parborell’s group demonstrated that C1P reduces ovarian injury during chemotherapy with alkylating agents (Pascuali et al., 2018) assigning a crucial role to the sphingolipid.

Our findings are laying the groundwork for additional research about the physiological Pg pathway that remains to be fully ascertained. This study uncovers the sphingolipids’ function during exocytosis, particularly, the role of the critical bioactive lipid C1P. Here, we emphasize the indispensable lipid balance required for fertilization. The data provided here may open novel scenarios about the pathways that could be affected in male infertility bringing basic knowledge closer to translational medicine.

## Data Availability

The original contributions presented in the study are included in the article/Supplementary Material, further inquiries can be directed to the corresponding author.
