# Supplementary material for: The pair ceramide 1-phosphate/ceramide kinase regulates intracellular calcium and progesterone-induced human sperm acrosomal exocytosis
Source: Front Cell Dev Biol. 2023 Mar 31;11:1148831. doi: 10.3389/fcell.2023.1148831 (PMC10102357; doi:10.3389/fcell.2023.1148831)
Supplement: Supplementary file 3 [file DataSheet1.PDF]

Supplementary movie 1

A B C

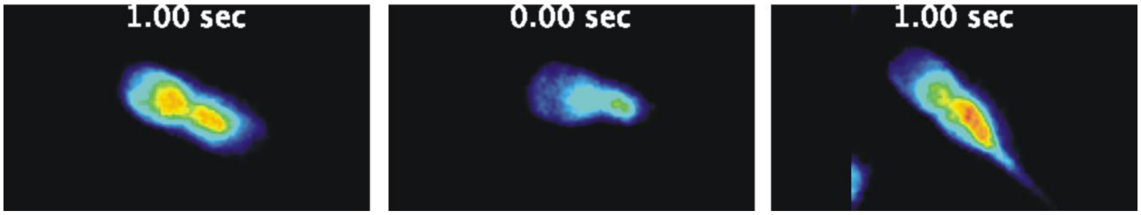

C1P-triggers intracellular calcium increase. Capacitated human sperm were loaded with Fluo-3 AM (2 μM) and suspended in HTF medium containing a final concentration of 2 mM $CaCl_2$. The fluorescence intensity was visualized before and after C1P addition as described under "Materials and Methods". These movies are a synchronized montage of fluorescence images of C1P treated human spermatozoa. Images of sperm incubated with 10 μM C1P, were originally recorded at 2 frames/sec. Color bar is the same as shown in Fig. 2A, 2B and 2C. See Fig. 2A, 2B and 2C for still images of movie sequences.
